# ψ-Bufarenogin, a novel anti-tumor compound, suppresses liver cancer growth by inhibiting receptor tyrosine kinase-mediated signaling

**DOI:** 10.18632/oncotarget.3435

**Published:** 2015-03-23

**Authors:** Jin Ding, Wen Wen, Daimin Xiang, Peipei Yin, Yanfang Liu, Chang Liu, Guoping He, Zhuo Cheng, Jianpeng Yin, Chunquan Sheng, Wen Zhang, Fajun Nan, Wencai Ye, Xiuli Zhang, Hongyang Wang

**Affiliations:** ^1^ The International Cooperation Laboratory on Signal Transduction, Eastern Hepatobiliary Surgery Hospital, Second Military Medical University, Shanghai, China; ^2^ Department of Pharmacology, Shanghai Institute of Pharmaceutical Industry, Shanghai, China; ^3^ Key Lab of Separation Science for Analytical Chemistry, Dalian Institute of Chemical Physics, Chinese Academy of Sciences, Dalian, China; ^4^ College of Pharmacy, Second Military Medical University, Shanghai, China; ^5^ National Center for Drug Screen, Shanghai, China; ^6^ College of Pharmacy, Jinan University, Guangzhou, China; ^7^ National Center for Liver Cancer, Shanghai, China

**Keywords:** ψ-Bufarenogin, hepatocellular carcinoma, epithelial growth factor receptor, hepatocyte growth factor receptor

## Abstract

Resistance of hepatocellular carcinoma (HCC) to existing chemotherapeutic agents largely contributes to the poor prognosis of patients, and discovery of novel anti-HCC drug is in an urgent need. Herein we report ψ-Bufarenogin, a novel active compound that we isolated from the extract of toad skin, exhibited potent therapeutic effect in xenografted human hepatoma without notable side effects. *In vitro*, ψ-Bufarenogin suppressed HCC cells proliferation through impeding cell cycle progression, and it facilitated cell apoptosis by downregulating Mcl-1 expression. Moreover, ψ-Bufarenogin decreased the number of hepatoma stem cells through Sox2 depression and exhibited synergistic effect with conventional chemotherapeutics. Mechanistic study revealed that ψ-Bufarenogin impaired the activation of MEK/ERK pathway, which is essential in the proliferation of hepatoma cells. ψ-Bufarenogin notably suppressed PI3-K/Akt cascade, which was required in ψ-Bufarenogin-mediated reduction of Mcl-1 and Sox2. ψ-Bufarenogin inhibited the auto-phosphorylation and activation of epithelial growth factor receptor (EGFR) and hepatocyte growth factor receptor (c-Met), thereafter suppressed their primary downstream cascades Raf/MEK/ERK and PI3-K/Akt signaling. Taken together, ψ-Bufarenogin suppressed HCC growth via inhibiting, at least partially, receptor tyrosine kinases-regulated signaling, suggesting that ψ-Bufarenogin could be a novel lead compound for anti-HCC drug.

## INTRODUCTION

With nearly 600,000 deaths per year, hepatocellular carcinoma (HCC) ranks as the second most common cause of cancer death worldwide [1]. HCC morbidity is essentially synonymous with mortality because the vast majority of HCC patients are diagnosed at a late stage when it is too far advanced to be cured. Surgical ablation is only effective in a minority of patients, and the effect of chemoembolization remains disappointing. Moreover, HCC is highly resistant to existing chemotherapeutic agents, no matter administered alone or in combination [2]. Sorafenib is considered to be the only effective targeted agent for clinical use in late-stage patients; nevertheless, its therapeutic effect is rather disappointing. Therefore, a novel anti-HCC drug is urgently needed.

To date, natural products or their derivatives have been one of the major sources of effective medicinal ingredients. Natural compounds possess not only great biological activity but also enormous structural diversity, and thus cellular biochemistry is hardly replaced by chemical synthesis despite the advances of modern chemistry [3]. More than half of currently available drugs are natural compounds or their derivatives, and the proportion surpasses 60% for cancer drugs [4]. Traditional Chinese medicines (TCMs) have been broadly recognized in China for many years for providing effective and safe cancer treatments, and these medicines are also considered as a valuable resource for novel anti-tumor lead compounds.

The skin of toad has been used in the treatment of liver cancer in Chinese medical practices since ancient times. Extract of toad skin is widely used as a TCM for treating advanced HCC in our and other hospitals in China at present [5]. However, the effective constituent of toad skin extract is unclear and the molecular mechanism underlying the therapeutic effect remains obscure, which hinders the recognition and popularization of toad skin extract in European and American countries. Moreover, severe side effects raised by non-effective components also restrict the application of toad skin extract to a large extent. Therefore, the further purification of toad skin and the illustration of related anti-HCC mechanisms are required. Previous phytochemical investigations indicated that the primary bioactive components of toad skin could be a type of steroids called bufadienolides, some of which exhibit cytotoxicity in cancer cells *in vitro* while arise serious adverse effects such as arrhythmia *in vivo* [6, 7]. Therefore, the isolation and identification of novel bioactive compounds with the fewest side effects from toad skin are necessary.

The discovery of active compounds from natural products is generally a time-consuming process. In recent years, we have developed a novel two-dimensional reversed-phase liquid chromatography/hydrophilic interaction chromatography (2D-RPLC/HILIC) system with a Click b-Cyclodextrin (Click-CD) stationary phase, which displayed excellent orthogonality and was successfully employed to separate the high and intermediate polarity components in TCM [8, 9]. Through bioassay-guided stepwise isolation, a set of compounds including Bufarenogin, ψ-Bufarenogin and Gamabufotalin *etc*. were successfully purified [8, 9], among which ψ-Bufarenogin displayed a potent suppressive effect on hepatoma cells and little cytotoxicity in normal cells. The anti-HCC capacity of ψ-Bufarenogin along with its underlying molecular mechanism was thus systematically investigated in the current study.

## RESULTS

### ψ-Bufarenogin suppresses xenografted HCC growth in mice

**Figure 1:**
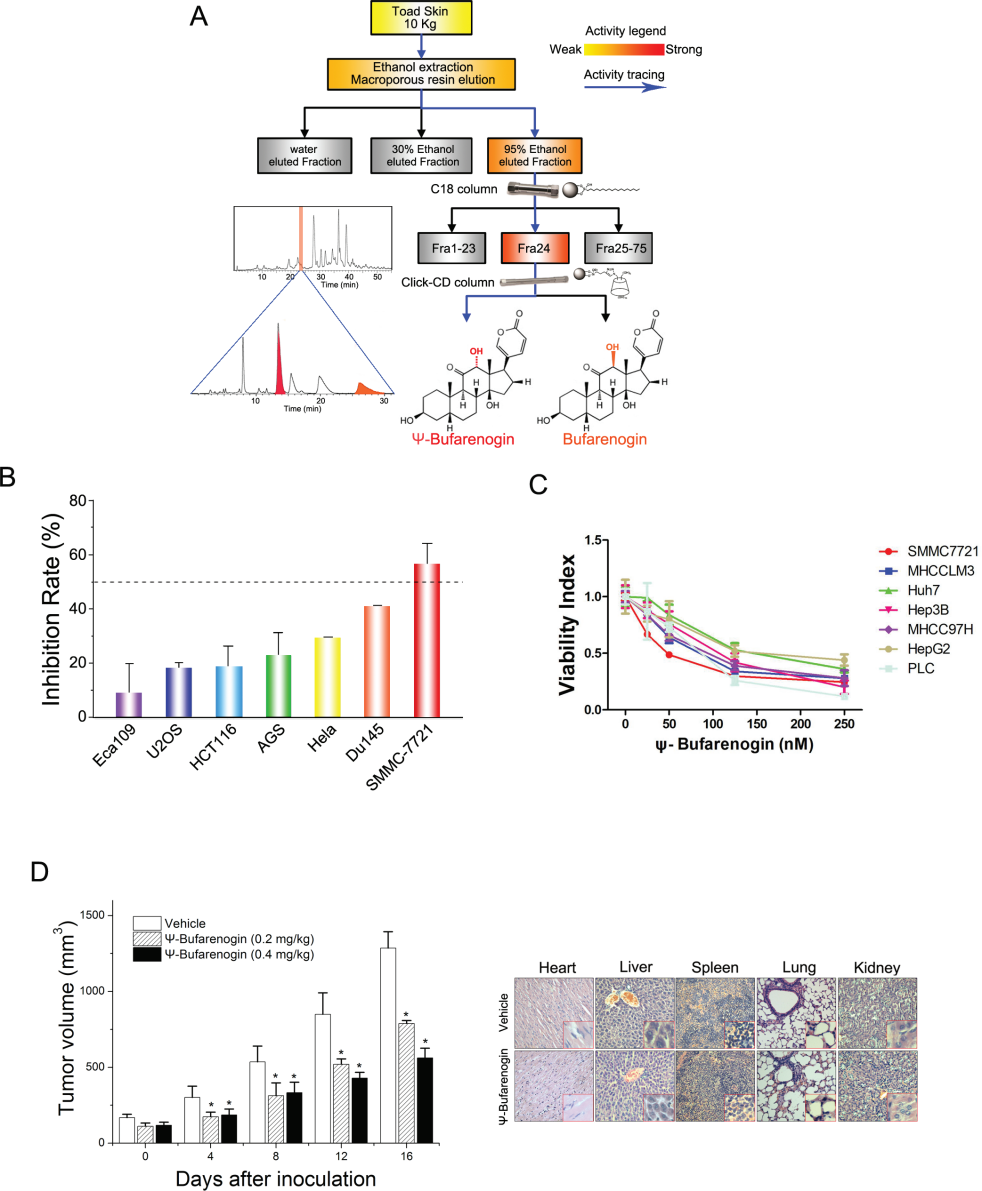
The therapeutic effect of ψ-Bufarenogin on liver cancer **(A)** A flow diagram of ψ-Bufarenogin isolation from toad skin using reversed-phase liquid chromatography coupled with hydrophilic interaction chromatography as previously described. **(B)** Seven cancer cell lines were exposed to 50 nM ψ-Bufarenogin for 48 hours. The cell viability was measured by CCK-8 assay and the inhibition rate was calculated. Data are represented as mean ± SEM. *p < 0.05. **(C)** Seven HCC cell lines were treated with 50 nM ψ-Bufarenogin for 48 hours followed by a cell viability assay. Data are represented as mean ± SEM. *p < 0.05. **(D)** SMMC-7721 cells-derived xenografts were implanted s.c. in the flanks of nude mice followed by i.v. administration of ψ-Bufarenogin. Tumor volume was monitored as described above (left). H&E staining of mice organs was performed after ψ-Bufarenogin therapy (right).

As shown in Figure 1A, Bufarenogin and ψ-Bufarenogin, the epimers at C-12, were isolated from toad skin through an integrated orthogonal isolation method [9]. Interestingly, ψ-Bufarenogin is nearly 100-fold more active than Bufarenogin against cancer cells, thereby Bufarenogin was excluded from further evaluation. The effects of ψ-Bufarenogin on the viability of cultured cancer cells from the most common cancer types were examined, and the result showed that HCC cell line SMMC-7721 was most sensitive to the compound (Figure 1B). ψ-Bufarenogin reduced the viability of all seven HCC cell lines in a dose-dependent manner (Figure 1C). The intratumoral injection of ψ-Bufarenogin significantly inhibited xenografted HCC growth in mice without undesirable adverse effects (Supplementary Figure 1A–1C). Consistent with this finding, the intravenous injection of ψ-Bufarenogin resulted in a dramatic reduction in the tumor volume up to 60% in comparison with that of the control group (Figure 1D&Supplementary Figure 1D). The vital organs and blood samples of treated mice were collected and subjected to cytotoxic side effect assessments. As shown in Figure 1D and Supplementary Table 1, ψ-Bufarenogin did not cause obvious side effect *in vivo*.

### ψ-Bufarenogin inhibits cell cycle progression and proliferation of HCC cells

**Figure 2:**
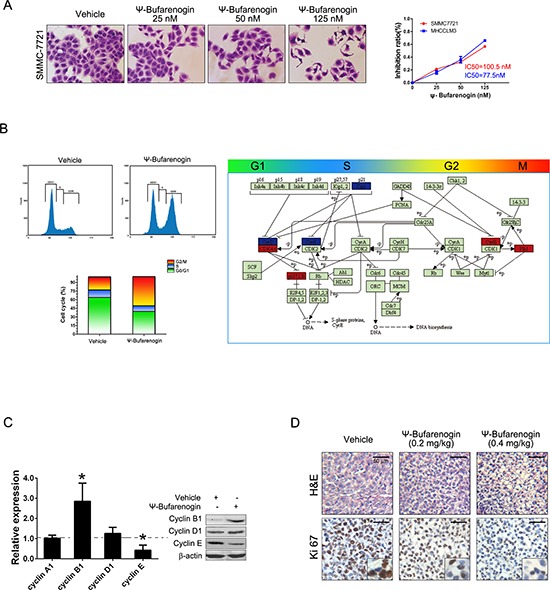
ψ-Bufarenogin suppresses hepatoma cells proliferation **(A)** SMMC-7721 and MHCC-LM3 cells were exposed to ψ-Bufarenogin at indicated dosages for 48 hours. CCK-8 assay was performed to calculate the inhibition rate and IC50. The cells were then stained with crystal violet and representative pictures were shown. **(B)** The cell cycle distribution of ψ-Bufarenogin-treated hepatoma cells was analyzed by flow cytometry (left). The differential expression of cell cycle-related genes in ψ-Bufarenogin-treated SMMC-7721 cells relative to control cells were achieved by Illumina microarray. Box in Red indicates upregulation and box in blue indicates downregulation (right). **(C)** The fold change of cyclin expression in ψ-Bufarenogin-treated SMMC-7721 cells relative to control cells was analyzed by real-time PCR. Western blot was conducted as described in Method. **(D)** The H&E staining and immunohistochemistry of Ki-67 antigen from xenografted hepatoma in mice treated with ψ-Bufarenogin (i.v.) as described above.

As shown in Figure 2A, ψ-Bufarenogin treatment inhibited the proliferation of HCC cells in a dose-dependent manner. Consistently, ψ-Bufarenogin-treated hepatoma cells generated fewer and smaller colonies in anchored or non-anchored condition (Supplementary Figure 2A & 2B). Flow cytometry data showed a decreased G1/S transition and a marked G2/M arrest in hepatoma cells exposed to ψ-Bufarenogin (Figure 2B). Expression profile of cell cycle-associated genes in the hepatoma cells exposed to ψ-Bufarenogin was analyzed by Illumina microarrays (Figure 2B). ψ-Bufarenogin-triggered reduction of cyclin E, a major mediator of G1/S transition, and the accumulation of cyclin B1, the destruction of which is required for anaphase onset (escape from mitosis), were validated (Figure 2C). Consistently, xenografts from ψ-Bufarenogin-treated mice displayed lower Ki67 antigen expression than those in the control group (Figure 2D).

### Mcl-1 reduction by ψ-Bufarenogin facilitates hepatoma cell apoptosis

**Figure 3:**
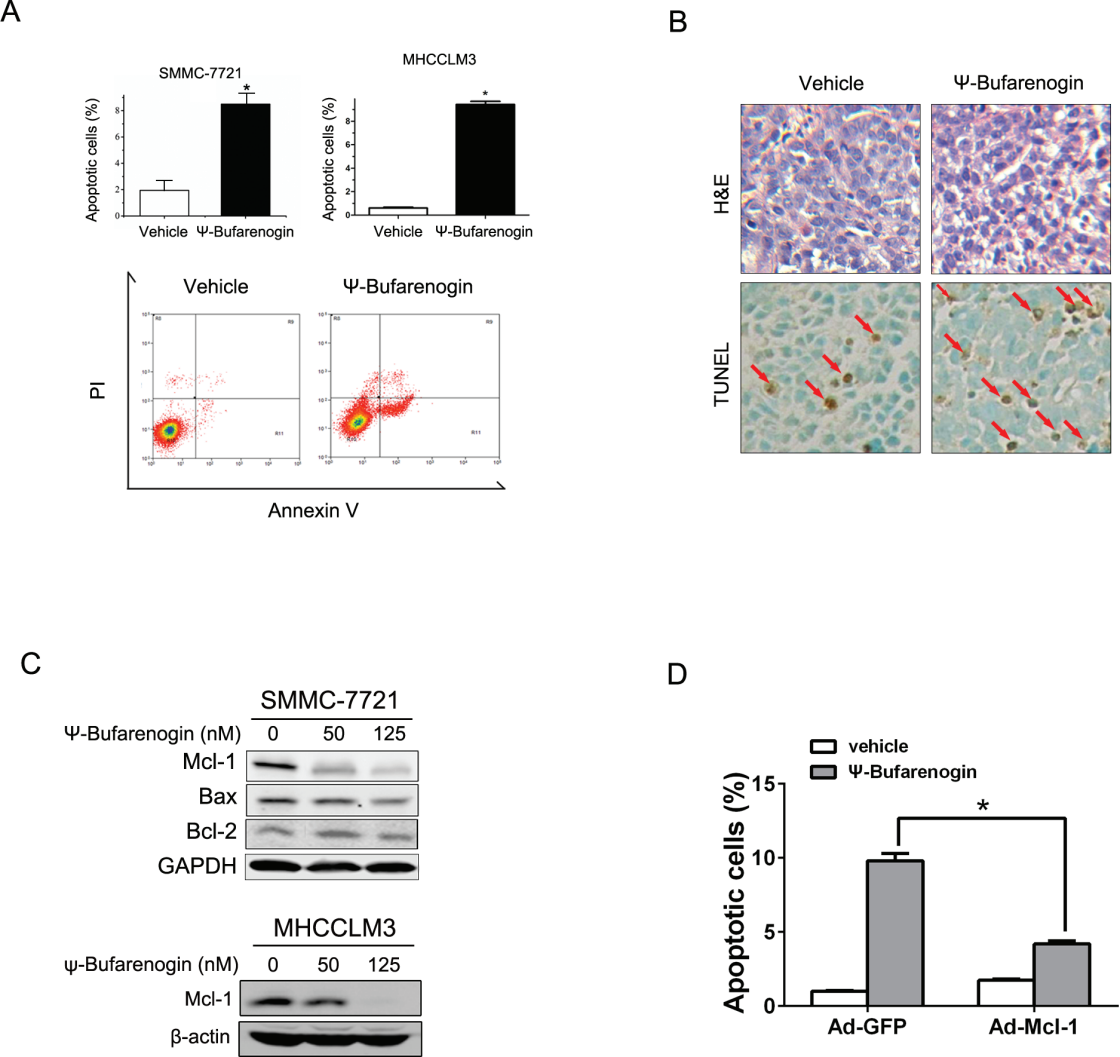
ψ-Bufarenogin promotes hepatoma cell apoptosis **(A)** Hepatoma cells were treated with 50 nM ψ-Bufarenogin for 48 hours. Apoptotic cells were determined by FACs. Data are represented as mean ± SEM. *p < 0.05. **(B)** The apoptotic cells in HCC xenografts from nude mice treated with ψ-Bufarenogin (i.v.) were determined by TUNEL assay. Red arrows indicate apoptotic cells. **(C)** Hepatoma cells were treated with ψ-Bufarenogin for 24 hours followed by western blot assay. **(D)** SMMC-7721 cells infected by Ad-GFP or Ad-Mcl-1 were treated with 50 nM ψ-Bufarenogin for 48 hours followed by FACs. Data are represented as mean ± SEM. *p < 0.05.

Hepatoma cells exposed to a high dosage of ψ-Bufarenogin experienced a dramatic increase in cell apoptosis (Figure 3A &Supplementary Figure 3A). More apoptotic cells were detected by TUNEL staining in HCC xenografts from ψ-Bufarenogin-treated mice in comparison with the control mice (Figure 3B). ψ-Bufarenogin enhanced cisplatin-triggered apoptosis of patient primary hepatoma cells and exhibited a synergistic effect when combined with cisplatin (Supplementary Figure 3B & 3C). Interestingly, ψ-Bufarenogin elicited a dose-dependent Mcl-1 reduction in hepatoma cells, but the levels of Bax and Bcl-2 were not remarkably altered (Figure 3C). Mcl-1 is an anti-apoptotic member of the Bcl-2 family and plays an essential role in the chemoresistance of HCC cells [10]. As expected, reconstitution of Mcl-1 expression robustly reduced the cell apoptosis triggered by ψ-Bufarenogin, suggesting that ψ-Bufarenogin facilitates the apoptosis of hepatoma cells through downregulating Mcl-1 expression (Figure 3D).

### ψ-Bufarenogin represses hepatic T-ICs expansion through Sox2 reduction

**Figure 4:**
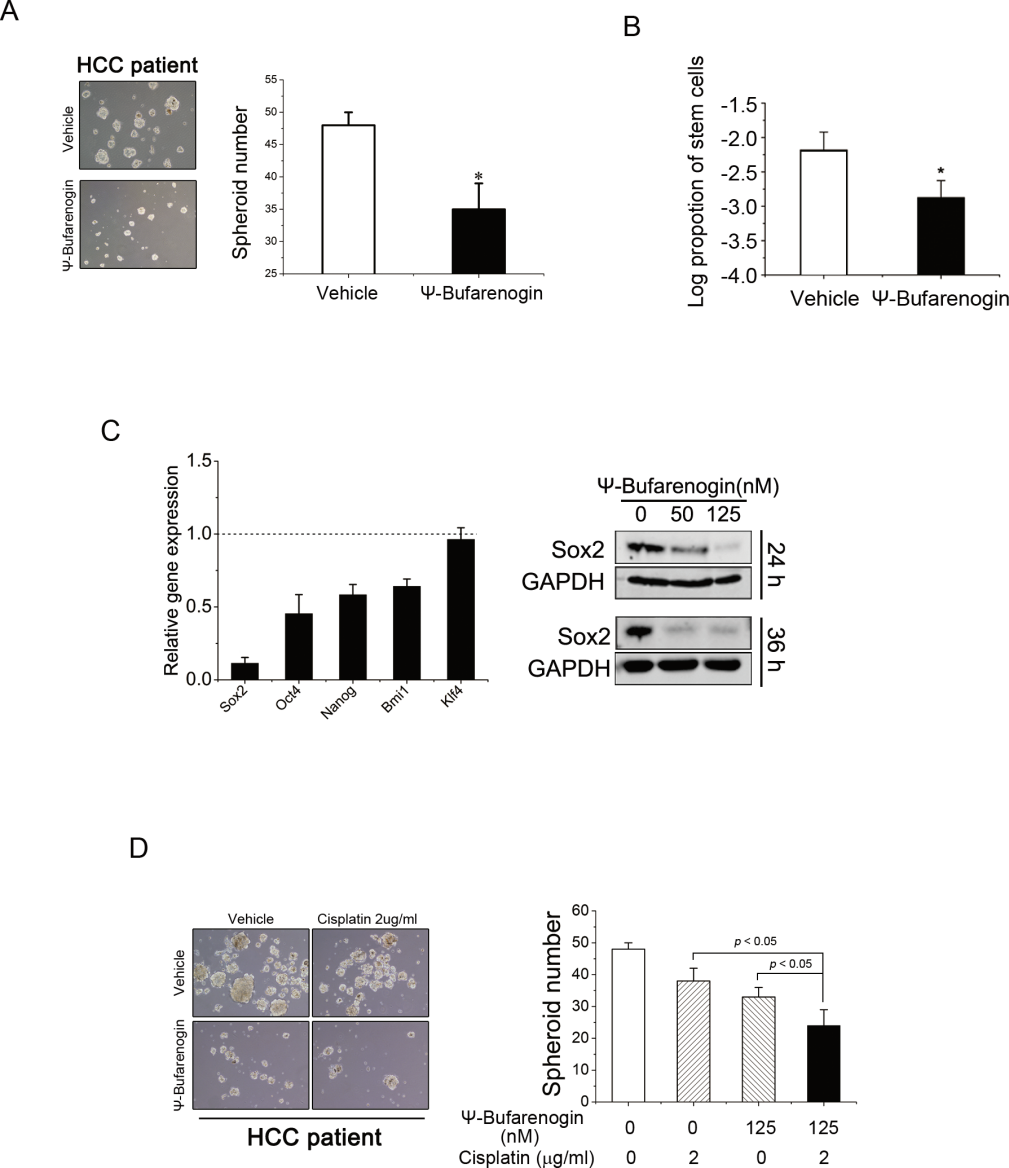
ψ-Bufarenogin inhibits the expansion of hepatic T-ICs **(A)** Spheroid formation assay of ψ-Bufarenogin (50 nM)-treated primary hepatoma cells isolated from patients. Data are represented as mean ± SEM. *p < 0.05. **(B)** Limiting dilution assay of SMMC-7721 cells exposed to ψ-Bufarenogin (50 nM), and the estimated proportion of cancer stem cells was shown as its natural logarithm. Data are represented as mean ± SEM. *p < 0.05. **(C)** Relative expression levels of Sox2, Oct4, Nanog, Bmi1 and Klf4 in SMMC-7721 cells treated with ψ-Bufarenogin in comparison with control cells. Data are represented as mean ± SEM. *p < 0.05. Western blot assay of Sox2 expression in SMMC-7721 cells exposed to ψ-Bufarenogin. **(D)** Spheroid formation assay of patient primary hepatoma cells treated with ψ-Bufarenogin and/or cisplatin. Data are represented as mean ± SEM. *p < 0.05.

Accumulating evidence indicated that HCC development was driven by a small population of cells termed tumor-initiating cells (T-ICs), which are capable of self-renewal and are resistant to conventional chemotherapy [11]. Herein we found that ψ-Bufarenogin markedly inhibited the expression of epithelial cellular adhesion molecule (EpCAM), CD133, and CD90, which are considered as biomarkers of hepatic T-ICs (Supplementary Figure 4A). ψ-Bufarenogin significantly inhibited the spheroid formation in HCC cell line and primary hepatoma cells of patients (Figure 4A & Supplementary Figure 4B). Moreover, the limiting dilution assay revealed that ψ-Bufarenogin significantly diminished the proportion of hepatic T-ICs in the whole cell population (Figure 4B & Supplementary Figure 4C). Self-renewal is regulated by a set of stemness-associated transcription factors, among which Sox2 expression was significantly downregulated by ψ-Bufarenogin in a dose-dependent manner (Figure 4C). We therefore hypothesized that ψ-Bufarenogin could chemosensitize HCC cells to chemotherapeutic agents by targeting the T-ICs. As expected, the combination of ψ-Bufarenogin and cisplatin exerted a synergistic suppression of the spheroid formation in HCC cell line and primary hepatoma cells of patients (Figure 4D & Supplementary Figure 4D).

### ψ-Bufarenogin inhibits the Raf/MEK/ERK and PI3-K/Akt pathways in HCC cells

**Figure 5:**
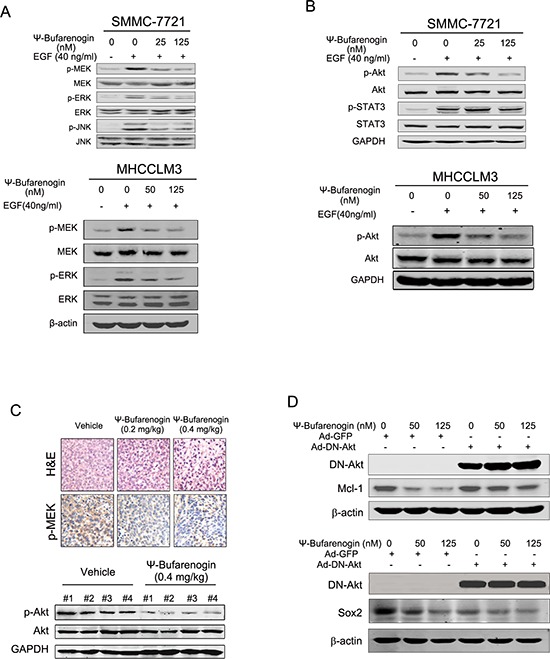
ψ-Bufarenogin suppresses Raf/MEK/ERKs and PI3-K/Akt cascades **(A & B)** Hepatoma cells pretreated with ψ-Bufarenogin as indicated were exposed to EGF for 15 min followed by western blot assay. **(C)** H&E staining and immunohistochemistry of p-MEK in the HCC xenografts of nude mice treated with ψ-Bufarenogin (i.v.). Representative pictures are shown. The extracts of HCC xenografts from nude mice described above were analyzed by western blot assay. **(D)** SMMC-7721 cells were infected by Ad-DN-Akt or Ad-GFP, and Mcl-1 and Sox2 expression was determined by western blot assay.

Activation of MAPKs pathway plays a pivotal role in the proliferation of cancer cells exposed to growth factors. An association between the anti-HCC activity of ψ-Bufarenogin and its influence on the MAPK pathway was therefore determined. As shown in Figure 5A, the activation of MEK/ERK cascade in HCC cells was significantly inhibited by ψ-Bufarenogin in a dose-dependent manner. ψ-Bufarenogin also slightly inhibited JNK phosphorylation, but it had no effect on p38 activation (Supplementary Figure 5A). PI3-K/Akt signaling is known to be involved in the survival of cancer cells and the self-renewal of T-ICs [12]. Therefore, the effect of ψ-Bufarenogin on Akt activation was examined. Pretreatment of ψ-Bufarenogin dramatically suppressed EGF-induced Akt phosphorylation in a dosage-dependent manner, while STAT3, a critical mediator of cell survival, was not influenced (Figure 5B). Consistently, suppression of p-MEK and p-Akt was observed in ψ-Bufarenogin-treated xenografted hepatoma in mice (Figure 5C). Moreover, the downregulation of Mcl-1 and Sox2 by ψ-Bufarenogin was markedly attenuated in hepatoma cells that were transfected with a dominant negative mutant of Akt, suggesting that Akt was involved in a ψ-Bufarenogin-mediated reduction of Mcl-1 and Sox2 (Figure 5D). Furthermore, we found that ψ-Bufarenogin inhibited the activation of Raf, the upstream modulator MEK/ERK signaling, in hepatoma cells (Supplementary Figure 5B). Akt activation is usually up-regulated by PI3-K and down-regulated by the PTEN tumor suppressor. Our data revealed that PTEN expression was not altered by ψ-Bufarenogin treatment, which excluded the involvement of PTEN (Supplementary Figure 5C), and a competitive ELISA assay demonstrated that ψ-Bufarenogin significantly repressed the kinase activity of PI3-K in hepatoma cells (Supplementary Figure 5D).

### ψ-Bufarenogin inhibits the activation of EGFR and c-MET in HCC cells

**Figure 6:**
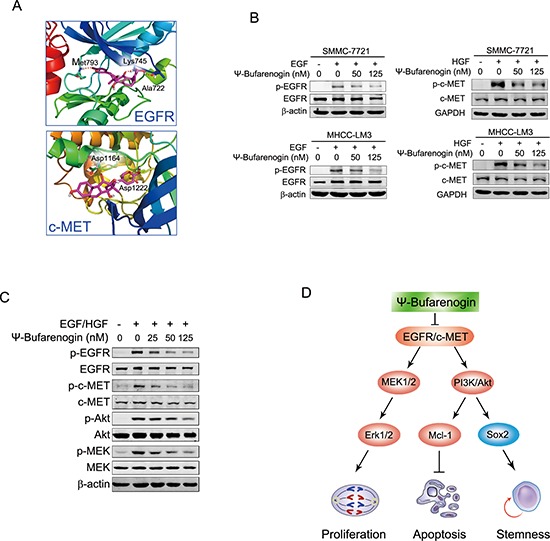
Dual inhibition of EGFR and c-MET by ψ-Bufarenogin in HCC **(A)** Binding model of ψ-Bufarenogin in the dimerization pocket of EGFR or c-MET. The position of hydrogen bonds between ψ-Bufarenogin (purple) and EGFR or c-MET was indicated. **(B)** The effect of ψ-Bufarenogin on the phosphorylation EGFR and c-MET in hepatoma cells was determined by western blot assay. **(C)** Western blot assay of EGFR and c-MET in addition to their downstream signaling molecules in EGF/HGF-stimulated SMMC-7721 cells pretreated with ψ-Bufarenogin. **(D)** A schematic representation of the mechanism underlying the inhibitory role of ψ-Bufarenogin against HCC progression.

Activation of MEK/ERK and PI3-K/Akt signaling are largely mediated by RTK, in particular EGFR and c-MET, in hepatoma cells. ψ-Bufarenogin was then docked into the crystal structure of EGFR and c-MET by computational modeling and the binding model was predicted by molecular docking (Figure 6A). ψ-Bufarenogin formed three major hydrogen bond interactions with EGFR. The carbonyl of the pyranone group made a hydrogen bond with the primary chain of the hinge region-Met793; the C-ring carbonyl group and terminal hydroxyl group formed hydrogen bonds with Lys745 and Ala722, respectively. As indicated in the interaction model of ψ-Bufarenogin and c-MET, the carbonyl of the pyranone group was positioned in the bottom of the cavity, forming a strong hydrogen bond with Asp1222; the C-ring carbonyl group formed a hydrogen bond with the backbone amide of Asp1164. Consistently, the phosphorylation of EGFR in hepatoma cells was significantly suppressed by ψ-Bufarenogin (Figure 6B). Likewise, ψ-Bufarenogin also blocked the phosphorylation of c-MET (Figure 6B & Supplementary Figure 6A), which are widely expressed in HCC cells and possess synergistic effect with EGFR [13, 14]. The phosphorylation of EGFR or c-MET has a profound effect on the full activation and biological function of these kinases [15]. As expected, the phosphorylation of MEK and Akt was inhibited in the same manner as that of EGFR and c-MET, suggesting that ψ-Bufarenogin suppresses HCC progression via the inhibition of EGFR and c-MET-mediated signaling (Figure 6C). Considering EGFR and c-MET are preferentially overexpressed in hepatoma cells rather than hepatocytes, selectivity of ψ-Bufarenogin was thereby evaluated. Distinct with conventional chemotherapeutics, ψ-Bufarenogin exhibited potent cytotoxicity against hepatoma cells, but modest activity in normal hepatocytes (Supplementary Figure 6B). ψ-Bufarenogin dramatically inhibited the growth of patient-derived xenografted HCC overexpressing EGFR and c-MET, implying its future application in personalized HCC treatment (Supplementary Figure 7), which is worthy of further investigation. Collectively, these data indicated that ψ-Bufarenogin suppressed HCC progression by, at least partially, inhibiting the RTKs-mediated signaling (Figure 6D).

## DISCUSSION

Resistance to conventional chemotherapy is one of the features of HCC, which contributes to the poor prognosis of patients [16]. Sorafenib is the only effective targeted drug in clinic so far, but its therapeutic effect is rather disappointing. The discovery of novel anti-HCC drugs remains in an urgent need. Combinatorial chemistry was once envisaged as a promising strategy to meet this demand [17]. Nevertheless, the limited library contents of chemistry-synthesized compounds could not meet the requirements for high throughput screens, and synthesized chemicals always exhibit cytotoxicity in normal cells. Therefore, only a few *de novo* combinatorial chemistry-created compounds have been approved for clinical use. Natural products, the other important resource for obtaining novel active compounds for drug development, have attracted more and more interest in recent decades. A good example is Taxol, which was isolated from the Pacific yew tree and was once hailed as the most important new cancer drug in decades. TCM, a safe and effective drug used in China for many years, is regarded as a valuable resource for novel lead compound of drug. The extract of toad skin has been widely applied as a TCM for HCC treatment since the Ming dynasty, but the effective constituent of toad skin extract remains unclear and the therapeutic effect lacks of scientific explanation. In our study, ψ-Bufarenogin was purified from toad skin through bioassay-guided stepwise isolation. We found that ψ-Bufarenogin suppressed HCC xenografts at very low dosages without notable side effect. Our data also showed that ψ-Bufarenogin acted as an RTK inhibitor and suppressed HCC progression via inhibiting, at least partially, the RTK-regulated signaling.

Proliferation and apoptosis are two critical hallmarks of tumor cells, and suppression of proliferation and induction of apoptosis are two principle mechanisms of anti-tumor drugs. In the present study, ψ-Bufarenogin slightly reduced the expression of cyclin E, which is an important mediator of G1/S phase transition. Intriguingly, ψ-Bufarenogin treatment led to a notable accumulation of cyclin B1 and G2/M cell cycle arrest. Cyclin B1 is well-established as a crucial regulator during cell mitosis, the destruction of which is indispensably required for anaphase onset (escape from mitosis) [18]. Therefore, cyclin B1 accumulation might be responsible, at least in part, for the ψ-Bufarenogin-triggered G2/M arrest of hepatoma cells. Apart from its cytostatic effect, ψ-Bufarenogin-triggered apoptosis was detected in hepatoma cells. Apoptosis is regulated by the balance between proapoptotic and antiapoptotic mediators. The Bcl-2 family of pro-survival and pro-apoptotic proteins including Bcl-2, Mcl-1 and Bax *etc*. play essential roles in regulating cell apoptosis. We found that Mcl-1 expression was dramatically reduced by ψ-Bufarenogin, and the expression of Bcl-2 and Bax was not obviously influenced. Reconstitution of Mcl-1 expression attenuated ψ-Bufarenogin-elicited apoptosis, indicating that ψ-Bufarenogin may facilitate hepatoma cell apoptosis via Mcl-1 reduction. Mcl-1 is highly expressed in a variety of human cancers, and it is closely associated with chemo-resistance. Mcl-1 has recently been recognized as a therapeutic target in cancer, and a related therapy has been tested in preclinical models [19]. Therefore, application of ψ-Bufarenogin might be extended to individualized medicine for patients with high Mcl-1 HCCs.

Accumulating studies support the concept that tumors are generated and maintained by a small, defined subset of cells termed “tumor initiating cells” or “cancer stem cells” [20, 21]. T-ICs are able to self-renew and are responsible for chemo-resistance and cancer recurrence. Substantial evidence has demonstrated that T-ICs exist in various tumors, including leukemia, glioma, breast cancer, *etc*. [22]. Liver T-ICs have also been identified by several cell surface antigens such as EpCAM, CD90, and CD133, *etc*. [23]. Targeting liver T-ICs is supposed to achieve long-lasting remission and even a cure for HCC. However, T-IC-targeted drugs have yet to be developed. In the present study, ψ-Bufarenogin repressed EpCAM, CD90, and CD133 expression and diminished hepatoma T-ICs in HCC cell line and primary hepatoma cells of patients. Self-renewal is usually regulated by a set of stemness-related mediators including Oct4, Sox2, Klf, Nanog and Bmi *etc*. [24]. Among these transcription factors, Sox2 expression was significantly down-regulated by ψ-Bufarenogin treatment, suggesting that Sox2 could be involved in the repressed expansion of hepatic T-ICs by ψ-Bufarenogin. Furthermore, the combination of ψ-Bufarenogin and cisplatin led to a synergistic effect in the HCC cell line and primary hepatoma cells from patients, implying that ψ-Bufarenogin might target liver T-ICs and facilitate the therapeutic effect of conventional chemotherapy.

The MAPK pathway is one of the most critical signaling cascades in HCC development, and the enhanced activation of MAPKs in HCC has been revealed in numerous studies. HCC treatment with MAPK signaling inhibitors including sorafenib has attracted tremendous interest [25]. In this study, ψ-Bufarenogin significantly depressed the activation of the Raf/MEK/Erks cascade, which could also be responsible for the suppressive effect of ψ-Bufarenogin on hepatoma cell proliferation. During the last ten years, the PI3-K/Akt pathway has emerged as an essential contributor to HCC development. Hepatoma exhibits the highest percentage of PIK3ca (p110 catalytic subunit of PI3-K) mutations (36%) among solid tumors, and sustained Akt activation leads to deleterious cell survival and chemo-resistance in HCC cells [26]. Activated Akt acted as an independent risk factor for HCC recurrence and for the poor prognosis of patients [27]. As shown in this work, ψ-Bufarenogin dramatically inhibited the activation of PI3-K/Akt signaling in hepatoma cells both *in vitro* and *in vivo*. Furthermore, reduction of Mcl-1 and Sox2 in hepatoma cells by ψ-Bufarenogin was robustly blocked by DN-Akt (the dominant negative mutant Akt) [27] overexpression, indicating that PI3-K/Akt should be involved in ψ-Bufarenogin-facilitated apoptosis of hepatoma cells and the repression of hepatic T-ICs expansion. Collectively, the suppression of both Raf/MEK/Erks and PI3-K/Akt signaling by ψ-Bufarenogin represented an attractive approach for HCC treatment.

Growth factors bind and interact with their tyrosine kinase receptors, including EGFR, HGFR, VEGFR, PDGFR *etc*., which in turn recruit and activate distinct downstream kinases such as Ras or PI3-K. Ras activation leads to a set of phosphorylation events involving Raf, MEK and ERK kinase, and PI3-K activated by RTKs or Ras triggers the phosphorylation of Akt [28]. Considering the inhibition of ψ-Bufarenogin on both MAPKs and PI3-K/Akt signaling, we speculated that ψ-Bufarenogin may influence RTK activation in hepatoma cells. EGFR is the most important RTK in cancer cells, and activated EGFR promotes tumor growth in numerous cancer types. The importance of EGFR in cancer progression has been validated by the clinical success of its targeted drugs, such as erlotinib and gefitinib *etc*. [29, 30]. Our data showed that the phosphorylation of EGFR and its downstream kinases were dramatically suppressed by ψ-Bufarenogin in hepatoma cells. The hyperactivation of c-MET is frequently observed in HCC cells and correlates with the poor prognosis of patients [31, 32]. c-MET cross-reacts and has a synergistic effect with EGFR and usually compensates for EGFR activity, thus conferring resistance to EGFR-targeting drugs. Nevertheless, there are few targeted drugs for clinical use that could inhibit EGFR and c-MET simultaneously at present. Herein we reported ψ-Bufarenogin strikingly inhibited the autophosphorylation and activation of both EGFR and c-MET. Taken together, these data suggested that ψ-Bufarenogin could be a promising drug candidate in HCC therapy particularly in the personalized treatment of HCCs in which EGFR/c-MET-driven signaling is indispensable for cancer progression. Moreover, ψ-Bufarenogin was well tolerated by host animals at therapeutically beneficial doses, making it a promising lead compound of anti-HCC drug for clinical trials.

## METHODS

### Compounds

Bufarenogin and ψ-Bufarenogin [C_24_H_32_O_6_] were isolated and purified from toad skin (*Bufo bufo gargarizans* Cantor skin) using a novel two-dimensional reversed-phase liquid chromatography/hydrophilic interaction chromatography (2D-RPLC/HILIC) system with a Click b-Cyclodextrin (Click-CD) stationary phase [9]. Compounds were dissolved in DMSO and diluted with normal sodium to the desired concentration for *in vitro* and *in vivo* studies.

### Cell lines and primary cells

Eca109, U2OS, HCT116, AGS, Hela and Du145 cancer cell lines and SMMC-7721, Huh7, Hep3B, HepG2, PLC, MHCC-97H and MHCC-LM3 hepatoma cell lines were cultured in DMEM (Invitrogen, Inc., Carlsbad, CA) supplemented with 1% L-glutamine, and 10% heat-inactivated FBS (Invitrogen, Inc.). The cancer cell lines used in the study were purchased from Cell Bank of Type Culture Collection of Chinese Academy of Sciences, Shanghai Institute of Cell Biology, Chinese Academy of Sciences, where they were characterized by cell vitality detection, DNA-Fingerprinting, isozyme detection and mycoplasma detection. These cell lines were immediately expanded and frozen so that they could be restarted every 3 to 4 months from a frozen vial of the same batch of cells. Primary hepatoma cells were isolated from HCC tissues taken from HCC patients who underwent curative resection at the Eastern Hepatobiliary Surgery Hospital (Shanghai, China) and the procedure was approved by the Ethics Committee of the Hospital.

### Real-time PCR and western blot

The original amount of specific transcripts was measured by real-time PCR with an ABI PRISM 7300 sequence detector (Applied Biosystems). The primer sequences are listed in Supplementary Table 2. Extracts of cell lysate or human HCC samples were analyzed by immunoblot with primary antibodies and IRDye 800CW-conjugated second antibody (LI-COR Biosciences). The antibodies are listed in Supplementary Table 3.

### Malignant behavior assays of hepatoma cells

The proliferation and cell cycle transition of hepatoma cells treated with ψ-Bufarenogin were determined as previously described [33]. The apoptosis of hepatoma cells triggered by ψ-Bufarenogin was examined by Vybrant Apoptosis Kit (Molecular Probes, Eugene, OR) and flow cytometry. To perform an anchor-independent growth assay, hepatoma cells were plated at 1 × 10^4^ cells per 60-mm dishes in DMEM containing 10% FBS and 30% (V/V) matrigel at the presence or absence of ψ-Bufarenogin. After 2 weeks, the multicellular colonies were counted under a microscope. For spheroid formation assay, primary hepatoma cells from patients were plated at 3 × 10^3^/ml in Corning 3261 ultra-low attachment culture dishes followed by ψ-Bufarenogin treatment. One week later, the spheroids formed were counted under the microscope. For limiting dilution assay, hepatoma cells were seeded into 96-well ultra-low attachment culture plates for 7 days. Spheroid formation was assessed by visual inspection. Based on the frequency of wells without colony, the proportion of stem cells was determined by using Poisson distribution statistics and L-Calc Version 1.1 software (Stem Cell Technologies, Inc., Vancouver, Canada) [34].

### Tumor xenograft experiments

Fragments of the SMMC-7721 xenograft were implanted subcutaneously into the flanks of nude mice. When the established tumors grew to ~300 mm^3^, the mice were randomly distributed into treatment and control groups. Intratumor injections of 0.5 mg/kg or 1.0 mg/kg ψ-Bufarenogin (*n* = 6) or vehicle were performed every other day. The tumor size was monitored every three days by using electronic calipers. Alternatively, ψ-Bufarenogin was given i.v. once daily at 0.2 or 0.4 mg/kg (*n* = 8) of ψ-Bufarenogin for 16 days. The tumor size was monitored every four days. For Patient-Derived HCC Xenograft (PDX) model, high expression of EGFR and c-MET in primary patient HCC was identified by western blot before mice inoculation. Intratumor injection of ψ-Bufarenogin (1.0 mg/kg) was conducted every the other day for 24 days. The tumor size was monitored as described above.

### Immunohistochemistry and TUNEL staining

Formaldehyde-fixed, paraffin-embedded sections of xenograft tumors were subjected to H&E staining and immunohistochemistry by following routine protocols. The antibody information is provided in Supplementary Table 3. Apoptotic cells in xenograft tumors from nude mice receiving ψ-Bufarenogin were detected *in situ* by TUNEL method by using a TdT-FragEL DNA Fragmentation Detection Kit (Oncogene, Boston, MA).

### PI3-K kinase activity assay

The PI3-K of EGF-stimulated SMMC-7721 cells with or without ψ-Bufarenogin pretreatment was immunoprecipitated with anti-p85α antibody and Protein A/G PLUS-Agarose beads (Santa Cruz Biotechnology). The PI3-K activity in the immunoprecipitates was measured by using a PI3-K enzyme-linked immunosorbent assay kit (Echelon Biosciences, Salt Lake City, UT) according to the manufacturer's instructions [35].

### Tyrosine phosphorylation assay of c-MET

SMMC-7721 cells preincubated with ψ-Bufarenogin for 4 h were stimulated with mitogen cocktail containing HGF for 7.5 min and cell lysate was extracted. Tyrosine phosphorylation of HGFR/c-MET was detected by a bead-based flow cytometric assay according to the instructions of the manufacturer's protocol (Novagen. Merck KGaA, Darmstadt, Germany).

### Molecular modeling

To investigate how ψ-Bufarenogin binds and inhibits EGFR and c-MET kinase, ψ-Bufarenogin was docked into the crystal structure of their kinase domains by computational modeling. The X-ray crystallographic structures of EGFR kinase domain in complex with the inhibitor (PDB: 3POZ [36]) and c-MET-inhibitor (PDB: 3zze [37]) were retrieved from the Protein Data Bank. The complexes were used as input for protein preparation by using the Discovery Studio 3.0 package (Discovery Studio Modeling Environment, Release 3.0. Accelrys Software Inc., San Diego). Hydrogen atoms were added to the complexes and water molecules were deleted beyond 5 Å from the het groups. The missing loops were then added by using the Prepare Protein protocol. The binding site was defined as whole residues within a 12 Å radius subset encompassing the ligands that were in complexes. Finally, all the water molecules were removed. Molecular docking was performed by using a CDOCKER protocol. Top hits and random conformations were both set to 20. CDOCKER energy and hydrogen bond interactions were considered as the criteria for choosing the top-ranked conformations of each docked complex.

### Statistical analysis

The significance of the difference between treatment and control groups was determined by using the Student's *t*-test, and statistical significance was set at *P* < 0.01 and *P* < 0.05.

## SUPPLEMENTARY FIGURES AND TABLES



## Abbreviations

HCC: hepatocellular carcinoma
TCM: traditional Chinese medicine
Mcl-1: Myeloid cell leukemia-1
T-IC: tumor-initiating Cell
Sox2: SRY-box containing gene 2
RTK: receptor tyrosine kinase
EGFR: epidermal growth factor receptor
c-MET: Mesenchymal-epithelial Transition Factor
