# Supplementary material for: ψ-Bufarenogin, a novel anti-tumor compound, suppresses liver cancer growth by inhibiting receptor tyrosine kinase-mediated signaling
Source: Oncotarget. 2015 Mar 23;6(13):11627–39. doi: 10.18632/oncotarget.3435 (PMC4484481; doi:10.18632/oncotarget.3435)
Supplement: Supplementary file 1 [file oncotarget-06-11627-s001.pdf]

SUPPLEMENTARY FIGURES AND TABLES

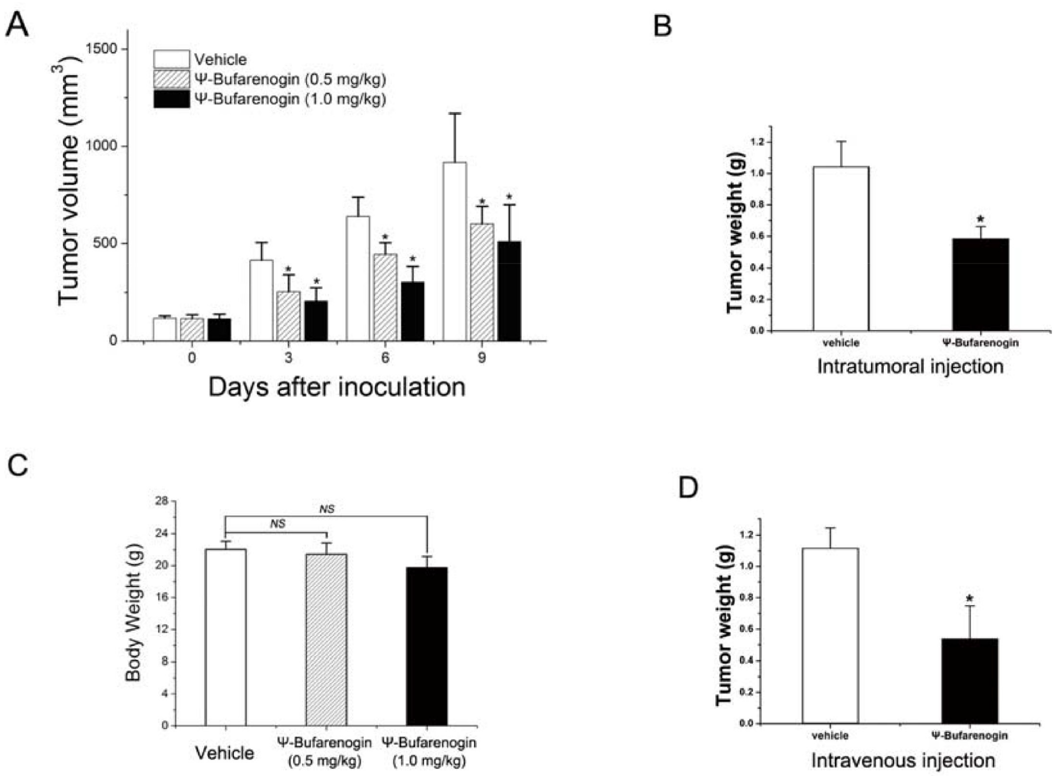

Supplementary Figure S1: (A) SMMC-7721 cells-derived xenografts were implanted s.c. in the flanks of nude mice followed by the intratumor injection of ψ-Bufarenogin. Tumor volume was measured by caliper. Data are represented as mean ± SEM. *$p < 0.05$. **(B)** SMMC-7721 cells-derived xenograft was transplanted s.c. into the flank of nude mice followed by intratumor injection of ψ-Bufarenogin. Xenograft tumors were resected and weighted after ψ-Bufarenogin treatment. Data are represented as mean ± SEM. *$p < 0.05$. **(C)** The body weights of the nude mice were measured after intratumor ψ-Bufarenogin administration. Data are represented as mean ± SEM. **(D)** SMMC-7721 cells-derived xenograft was transplanted s.c. into the flank of nude mice followed by i.v. administration of ψ-Bufarenogin. Xenograft tumors were resected and weighted after ψ-Bufarenogin treatment. *$p < 0.05$. Data are represented as mean ± SEM.

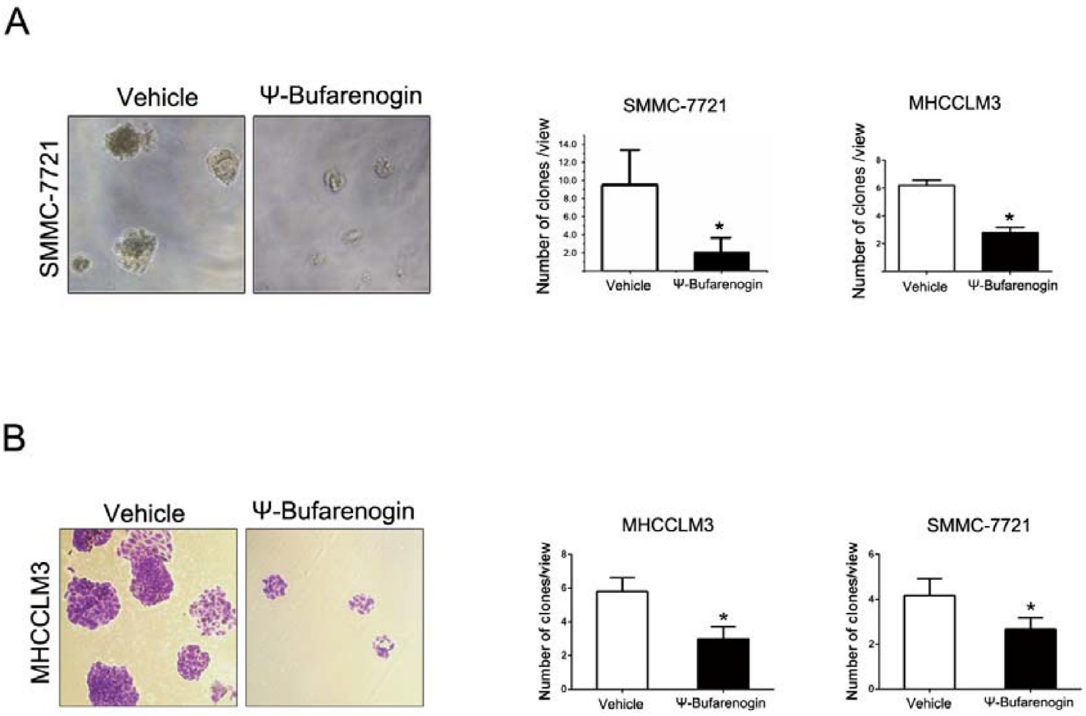

Supplementary Figure S2: (A) The anchor-independent growth of hepatoma cells exposed to ψ-Bufarenogin in matrigel were counted and compared. Data are represented as mean ± SEM. **p* < 0.05. **(B)** Colony formation in hepatoma cells treated with ψ-Bufarenogin on culture plates was measured after crystal violet staining. Data are represented as mean ± SEM. **p* < 0.05.

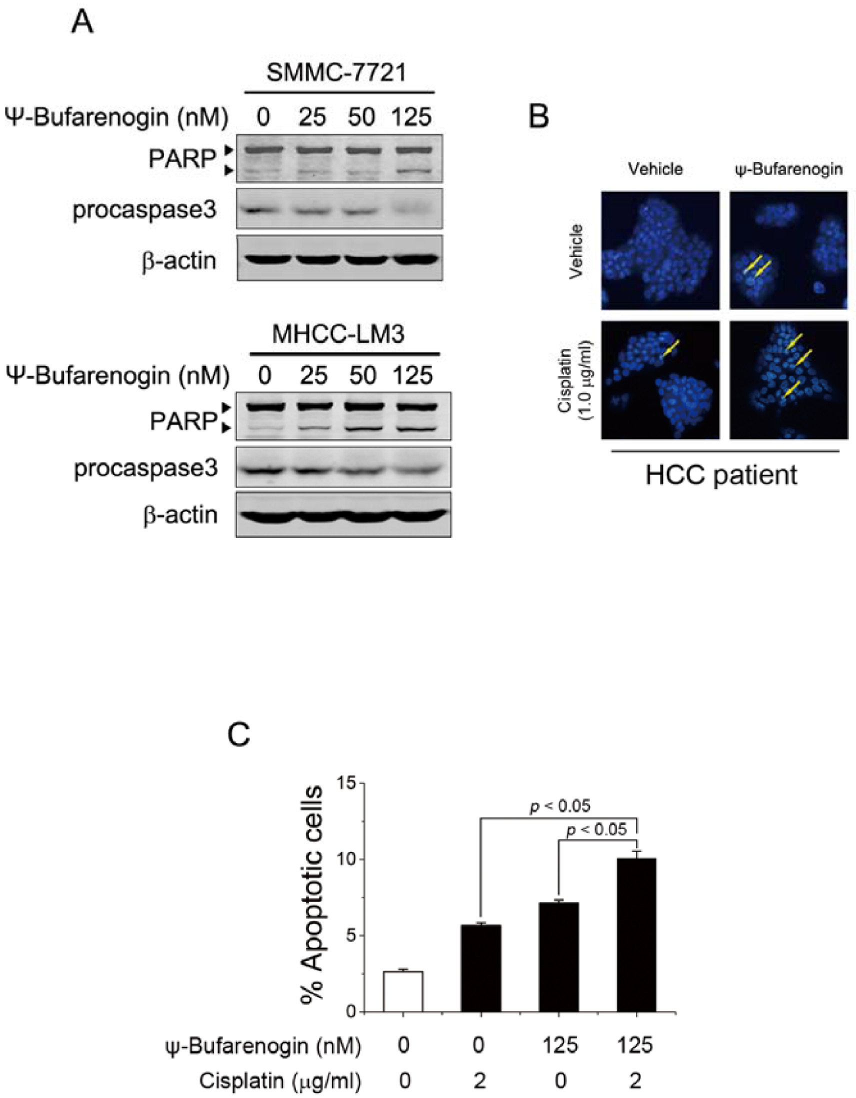

Supplementary Figure S3: (A) Western blot analysis of hepatoma cells treated with ψ-Bufarenogin for 48 hours. **(B)** Primary cultured hepatoma cells from fresh patient HCC tissues were treated with ψ-Bufarenogin and/or cisplatin as indicated. The cells were fixed with 4% polyformaldehyde and stained with PI. The yellow arrows indicate apoptotic cells. **(C)** Primary cultured hepatoma cells were treated with ψ-Bufarenogin and/or cisplatin for 48 hours. Apoptotic cells were measured by FACs. Data are represented as mean ± SEM.

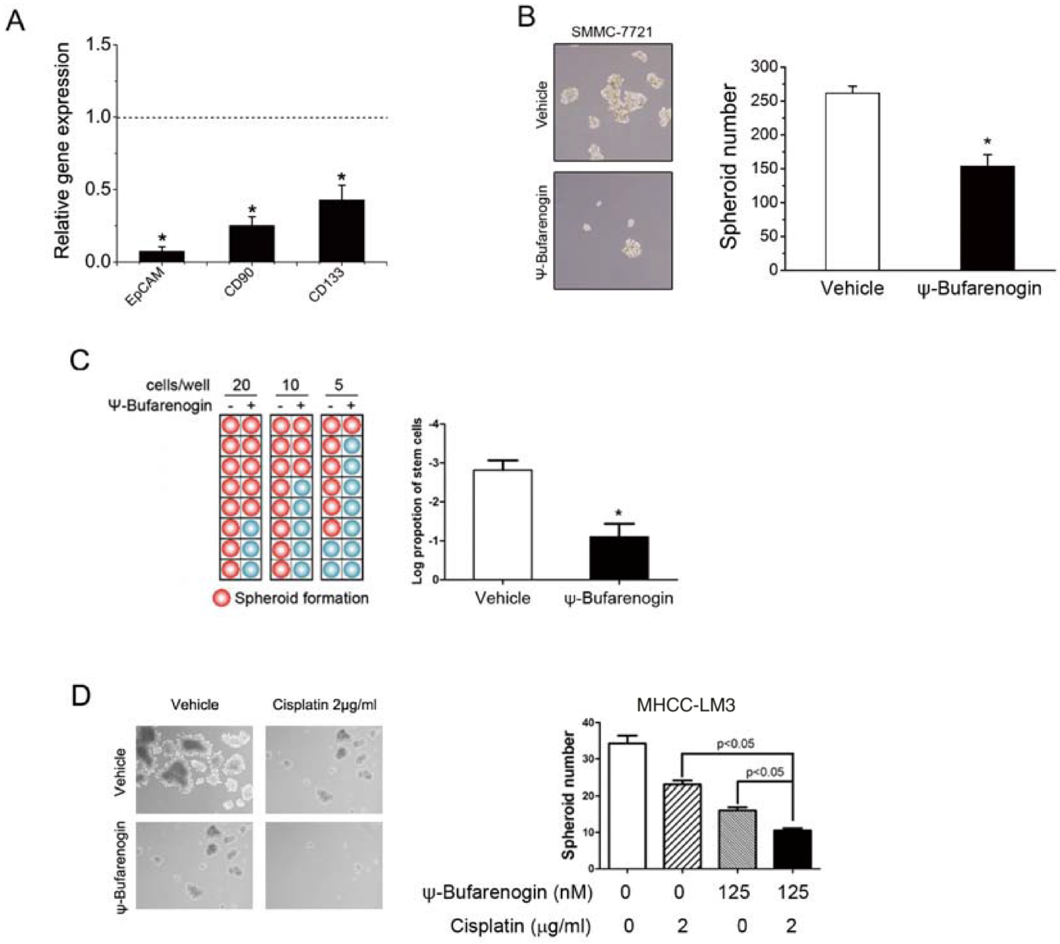

Supplementary Figure S4: **(A)** Relative expression levels of EpCAM, CD90 and CD133 in ψ-Bufarenogin-treated SMMC-7721 cells compared with control cells were analyzed by real-time PCR. Data are represented as mean ± SEM. **(B)** Spheroid formation assay of SMMC-7721 cells exposed to 50 nM ψ-Bufarenogin or vehicle control. Data are represented as mean ± SEM. *$p < 0.05$. **(C)** Limiting dilution assay of MHCC-LM3 cells exposed to ψ-Bufarenogin (50 nM), and the estimated proportion of cancer stem cells was shown as its natural logarithm. The red balls represent wells with spheroid formations. Data are represented as mean ± SEM. *$p < 0.05$. **(D)** Spheroid formation assay of hepatoma cells treated with ψ-Bufarenogin and/or cisplatin. Data are represented as mean ± SEM. *$p < 0.05$.

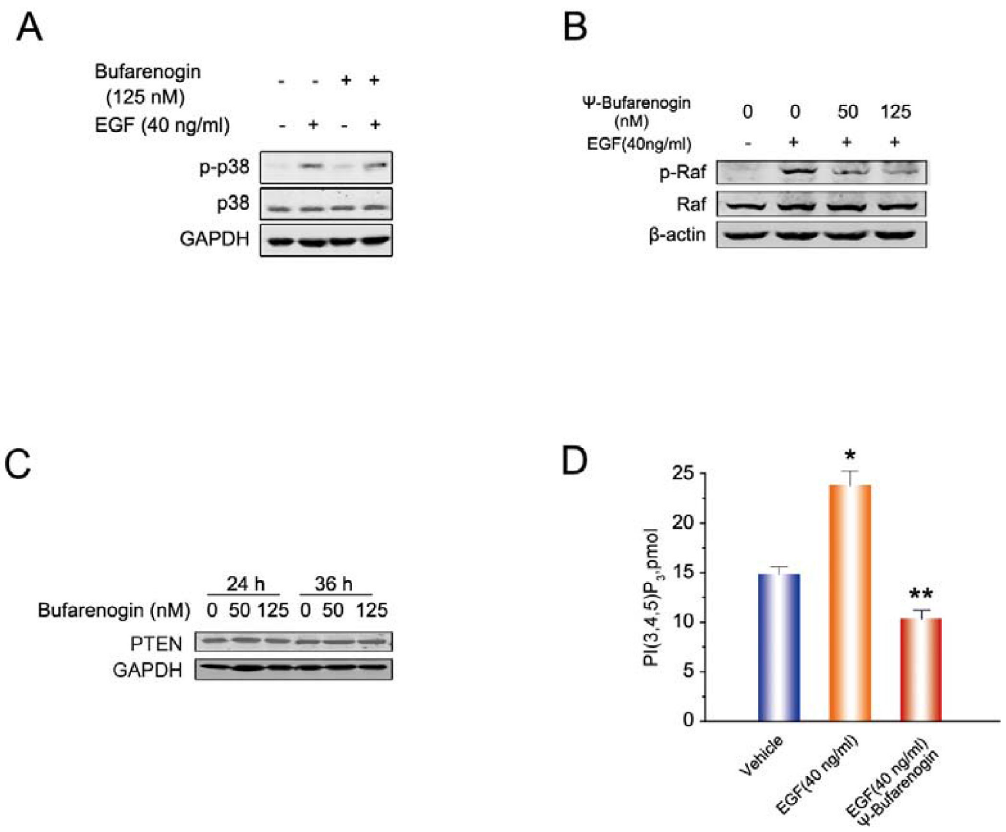

Supplementary Figure S5: (A&B) SMMC-7721 cells were pretreated with ψ-Bufarenogin for 4h and exposed to EGF for 15 min followed by western blot assay. **(C)** SMMC-7721 cells were exposed to ψ-Bufarenogin as indicated followed by western blot assay. **(D)** SMMC-7721 cells pretreated with ψ-Bufarenogin were exposed to EGF. Activation of PI3-K was measured by kinase activity assay. Data are represented as mean ± SEM. *$p < 0.05$.

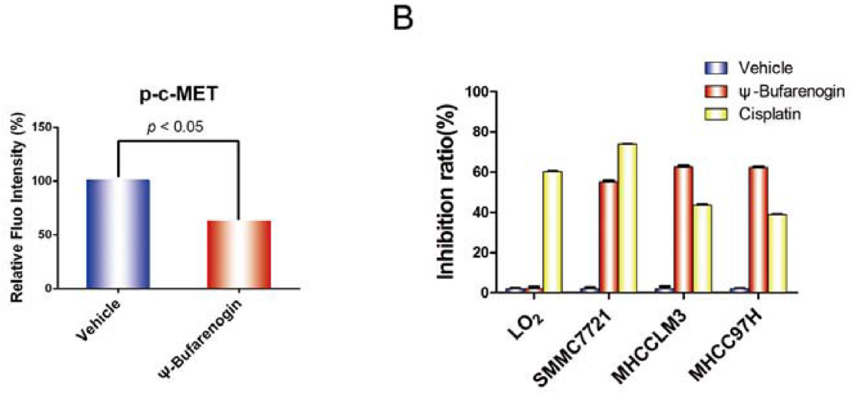

Supplementary Figure S6: (A) The effect of ψ-Bufarenogin on c-MET phosphorylation was determined by WideScreen™ RTK pTyr Assay (Merck. Inc.). **(B)** CCK8 assay of L02 normal hepatocytes and HCC cells treated with ψ-Bufarenogin (25 nM) and cisplatin (1 µg/ml). The inhibition ratio was calculated as described above. Data are represented as mean ± SEM.

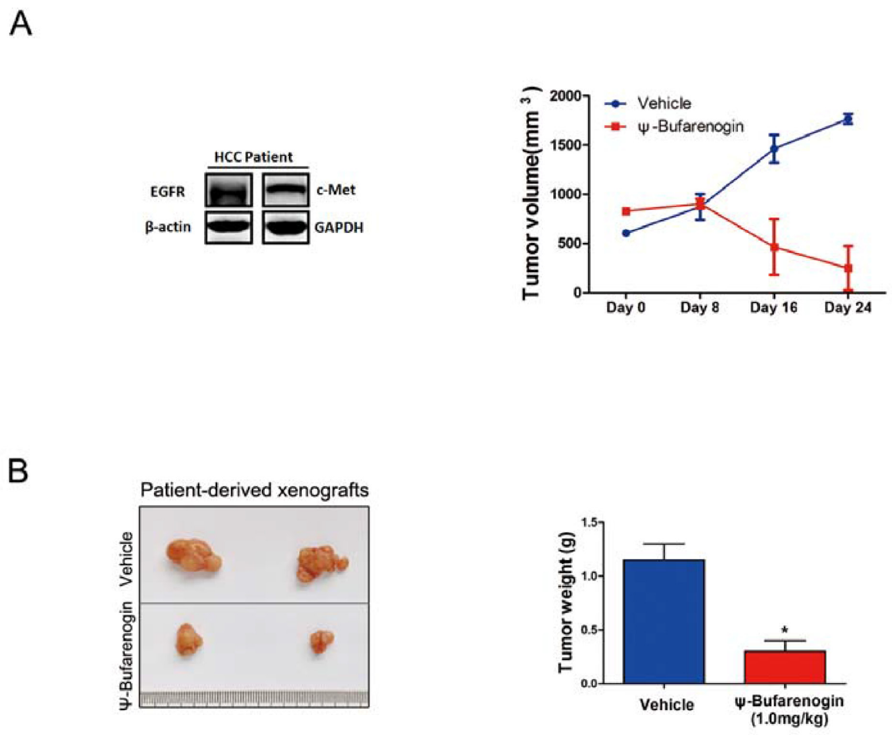

Supplementary Figure S7: (A) Western blot was performed to determine the expression of EGFR and c-MET in HCC patient-derived xenograft. Patient-derived xenograft was then transplanted s.c. into the flank of nude mice followed by intratumor injection (1 mg/kg) of ψ-Bufarenogin for 24 days. Tumor volume was measured as described above. **(B)** Xenograft tumors were resected and weighted after ψ-Bufarenogin treatment. Data are represented as mean ± SEM. *$p < 0.05$.

Supplementary Table S1: The function index of liver and kidney of mice (Mean ± SD)

Group	TP(IU/L)	ALB(IU/L)	GLB(IU/L)	A/G(IU/L)	ALT(IU/L)	AST(IU/L)	BUN(IU/L)	Cr(IU/L)
**vehicle**	60.96 ± 2.08	31.56 ± 1.51	30.00 ± 2.00	1.08 ± 0.109	71.35 ± 5.96	562.08 ± 33.96	9.00 ± 0.20	14.66 ± 1.15
**Bufarenogin (0.2 mg/kg)**	61 ± 3.38	33.6 ± 1.64	27 ± 2.58	1.275 ± 0.095	58.13 ± 7.53	561.33 ± 76.87	10.48 ± 0.87	21.5 ± 1.00

Supplementary Table S2: Sequence of primers for real-time PCR

Primer	Sequence (5′to3′)
Cyclin A1 forward primer	GTCAGAGAGGGGATGGCAT
Cyclin A1 reverse primer	CCAGTCCACCAGAATCGTG
Cyclin B1 forward primer	AAGAGCTTTAAACTTTGGTCTGGG
Cyclin B1 reverse primer	CTTTGTAAGTCCTTGATTTACCATG
Cyclin D1 forward primer	GCGCGCCCTCCGTTTCTTACTT
Cyclin D1 reverse primer	AGCTGCAGGCGGCTCTTCTT
Cyclin E1 forward primer	ATACAGACCCACAGAGACAG
Cyclin E1 reverse primer	TGCCATCCACAGAAATACTT
EpCAM forward primer	TCGCGTTCGGGCTTCTGCTT
EpCAM reverse primer	GGGCCCCTTCAGGTTTTGCT
CD 90 forward primer	CGGAAGACCCCAGTCCA
CD 90 reverse primer	ACGAAGGCTCTGGTCCACTA
CD133 forward primer	GCAGCAGTCTGACCAGCGTGAA
CD133 reverse primer	ACGGGTGGAAGCTGCCTCAGTT
Sox2 forward primer	AAATGGGAGGGGTGCAAAAGAGGAG
Sox2 reverse primer	CAGCTGTCATTTGCTGTGGGTGATG
Oct4 forward primer	CTTGCTGCAGAAGTGGGTGGAGGAA
Oct4 reverse primer	CTGCAGTGTGGGTTTCGGGCA
Nanog forward primer	AATACCTCAGCCTCCAGCAGATG
Nanog reverse primer	TGCGTCACACCATTGCTATTCTTC
Bmi1 forward primer	TGGAGAAGGAATGGTCCACTTC
Bmi1 reverse primer	GTGAGGAAACTGTGGATGAGGA
Klf4 forward primer	CCCACACAGGTGAGAAACCT
Klf4 reverse primer	ATGTGTAAGGCGAGGTGGTC
VEGF forward primer	CCTTGCTGCTCTACCTCCAC
VEGF reverse primer	ATCTGCATGGTGATGTTGGA
Beta-actin forward primer	AATCGTGCGTGACATTAAGGAG
Beta-actin reverse primer	ACTGTGTTGGCGTACAGGTCTT

Supplementary Table S3: Antibodies used for immunoblotting and immunohistochemistry

Protein	Antibody	Epitope mapping	Manufacturer
cyclin B1	Mouse monoclonal	Full length	Cell Signaling Technology Inc., Beverly, MA
cyclin D1	Rabbit monoclonal	Full length	Cell Signaling Technology Inc., Beverly, MA
cyclin E	Mouse monoclonal	Full length	Cell Signaling Technology Inc., Beverly, MA
PTEN	Rabbit polyclonal	Full length	ProteinTech Group, Inc. USA
Phospho-Akt	Rabbit monoclonal	Serine 473	Epitomics, Inc., Burlingame, CA
Akt	Rabbit polyclonal	C-terminus	Cell Signaling Technology, Beverly, MA, USA
Phospho-ERK	Rabbit monoclonal	Thr202/Tyr204	Cell Signaling Technology, Beverly, MA, USA
ERK	Rabbit monoclonal	C-terminus	Cell Signaling Technology, Beverly, MA, USA
GAPDH	Mouse monoclonal	Full length	Kangchen, Shanghai, P.R. China
Φ-actin	Rabbit polyclonal	C-terminus	Santa Cruz Biotechnology, Santa Cruz, CA
Phospho-MEK	Rabbit monoclonal	Ser217/221	Cell Signaling Technology, Beverly, MA, USA
MEK	Rabbit polyclonal	Full length	Cell Signaling Technology, Beverly, MA, USA
Phospho-JNK	Rabbit monoclonal	Thr183/Tyr185	Cell Signaling Technology, Beverly, MA, USA
JNK	Rabbit polyclonal	Full length	Cell Signaling Technology, Beverly, MA, USA
Phospho-STAT3	Rabbit monoclonal	Tyr705	Cell Signaling Technology, Beverly, MA, USA
STAT3	Mouse monoclonal	Full length	Cell Signaling Technology, Beverly, MA, USA
Mcl-1	Rabbit monoclonal	Full length	Cell Signaling Technology, Beverly, MA, USA
Bax	Mouse monoclonal	amino acids 3–16	Santa Cruz Biotechnology, Inc
Bcl-2	Mouse monoclonal	amino acids 1–205	Santa Cruz Biotechnology, Inc
CD34	Rabbit monoclonal	C-terminus	Epitomics, Inc., Burlingame, CA
Sox-2	Goat polyclonal	Full length	Abclonal, Shanghai, P.R. China
Phospho-EGFR	Rabbit monoclonal	Tyr1068	Epitomics, Inc., Burlingame, CA
EGFR	Rabbit monoclonal	Full length	Epitomics, Inc., Burlingame, CA
Phospho-c-MET	Rabbit polyclonal	Tyr1234	Sangon, Shanghai, P.R. China
c-MET	Rabbit polyclonal	Full length	Sangon, Shanghai, P.R. China
